# Antibiotic prescriptions in acute otitis media and pharyngitis in Italian pediatric outpatients

**DOI:** 10.1186/s13052-019-0696-9

**Published:** 2019-08-17

**Authors:** E. Barbieri, D. Donà, A. Cantarutti, R. Lundin, A. Scamarcia, G. Corrao, L. Cantarutti, C. Giaquinto

**Affiliations:** 1 Department for Woman and Child Health, University of Padua, Padua, Italy; 2 Division of Paediatric Infectious Diseases, Department for Woman and Child Health, University of Padua, Via Giustiniani 3, 35141 Padua, Italy; 3 PENTA Foundation, Padua, Italy; 4 National Centre for Healthcare Research and Pharmacoepidemiology, University of Milano-Bicocca, Milan, Italy; 5 Department of Statistics and Quantitative Methods, Unit of Biostatistics Epidemiology and Public Health, University of Milano-Bicocca, Milan, Italy; 6 Pedianet Project, Padua, Italy

**Keywords:** “Antibiotic prescriptions”, “wait and see”, “acute otitis media” “pharyngitis”, “Italian study”, “Pediatric patient”, “Pharmacoepidemiology”

## Abstract

**Background:**

Acute otitis media (AOM) and pharyngitis are very common infections in children and adolescents. Italy is one of the European countries with the highest rate of antibiotic prescriptions. The aim of this study is to describe first-line treatment approaches for AOM and pharyngitis in primary care settings in Italy over six years, including the prevalence of ‘wait and see’ for AOM, where prescription of antibiotics is delayed 48 h from presentation, and differences in prescribing for pharyngitis when diagnostic tests are used.

**Methods:**

The study is a secondary data analysis using Pedianet, a database including data at outpatient level from children aged 0–14 in Italy. Prescriptions per antibiotic group, per age group and per calendar year were described as percentages. “Wait and see” approach rate was described for AOM and pharyngitis prescriptions were further grouped according to the diagnostic test performed and test results.

**Results:**

We identified 120,338 children followed by 125 family pediatricians between January 2010 and December 2015 for a total of 923,780 person-years of follow-up. Among them 30,394 (mean age 44 months) had at least one AOM diagnosis (*n* = 54,943) and 52,341 (mean age 5 years) had at least one pharyngitis diagnosis (*n* = 126,098). 82.5% of AOM diagnoses were treated with an antibiotic within 48 h (mainly amoxicillin and amoxicillin/clavulanate) and the “wait and see” approach was adopted only in 17.5% of cases. The trend over time shows an increase in broad spectrum antibiotic prescriptions in the last year (2015). 79,620 (63%) cases of pharyngitis were treated and among GABHS pharyngitis confirmed by rapid test 56% were treated with amoxicillin. The ones not test confirmed were treated mainly with broad spectrum antibiotics.

**Conclusions:**

Despite guidance to use the ‘wait and see’ approach in the age group analyzed, this strategy is not often used for AOM, as previously noted in other studies in hospital settings. Broad-spectrum antibiotic prescription was more frequent when pharyngitis was not confirmed by rapid test, in keeping with evidence from other studies that diagnostic uncertainty leads to overuse of antibiotics.

**Electronic supplementary material:**

The online version of this article (10.1186/s13052-019-0696-9) contains supplementary material, which is available to authorized users.

## Background

Antimicrobials are the most widely prescribed drugs in children worldwide, both in hospital [1, 2] and community settings [3], especially at preschool age [4]. It has been estimated that 37–61% of hospitalized infants and children receive antibiotics [5–9] and almost half of antibiotic prescriptions in children are unnecessary [10–12]. Despite national and international efforts to promote appropriate antibiotic prescribing [13], Italy is one of the European countries with the highest rate of inappropriate antibiotic prescriptions (e.g. first line treatment prescription not in line with the guidelines or antibiotics prescribed for a diagnosis with a viral etiology), with an overuse of broad spectrum antibiotics [14–17].

Acute otitis media (AOM) and pharyngitis are two of the most common infections in pediatrics, and a main cause of antibiotic prescriptions [18].

AOM is an acute inflammation of the middle ear caused by viral (such as respiratory syncytial virus, rhinovirus, influenza viruses, and adenoviruses) or bacterial (such as *Streptococcus pneumoniae, non-typeable Haemophilus influenzae,* and *Moraxella catarrhalis*) infections [19]. AOM incidence in pediatric patients in Italy is estimated to be 16.8% [20] compared to worldwide incidence estimate of 10.85% [21].

National and international guidelines differentiate AOM treatment based on symptoms and the child’s age. The Italian society of Pediatrics (“Società Italiana di Pediatria” – SIP) [22] and the Italian Federation of Pediatricians (“Federazione Italiana Medici Pediatri” - FIMP) [23] guidelines support the “wait and see” approach when appropriate, and when antibiotic choice is recommended, amoxicillin is designed as the first drug of choice. Antibiotic therapy should be immediately administered if the child is less than 6 months of age, while it could be delayed in older children.

Acute pharyngitis is also a very common pediatric diagnosis, and *Group A β-hemolytic streptococcus* (GABHS) causes 37% of cases of pharyngitis in children older than 3 years [24, 25]. Indeed, despite the percentage of GABHS pharyngitis is around 25–30%, the antibiotic prescription rate appear to be from 10 to 20% higher [26, 27] .

According to international guidelines [28–31] and the national family pediatricians consensus document [23], first line treatment for GABHS pharyngitis is either amoxicillin or penicillin V since GABHS remains universally susceptible to penicillins. The rapid diagnostic test is highly recommended for all the children with clinical symptoms of GABHS pharyngitis [30] since its high sensitivity and specificity had been proved in various studies [32].

In Italy, pediatricians (FP) follow children aged 0–6 at which point families choose whether an FP or general practitioner (GP) follows the child to 14 years of age.

Considering the high prevalence of these infections in the pediatric population, the aim of this study is to describe the first-line treatment approach for AOM and pharyngitis at primary care level in Italian children across different age groups and calendar time, considering the ‘wait and see’ approach for AOM and the use and results of rapid testing for pharyngitis.

## Methods

### Study design

This observational, retrospective, outpatient study used an established Italian network of FPs (Pedianet) from January 2010 to December 2015 for the assessment of first line treatment of AOM and pharyngitis.

### Data source

Pedianet (http://www.pedianet.it), a pediatric general practice research database, contains reason for accessing healthcare, health status (according to the Guidelines of Health Supervision of the American Academy of Pediatrics), demographic data, diagnosis and clinical details (free text or coded using the 9th International Statistical Classification of Diseases and Related Health Problems system- ICD-9 CM), prescriptions (pharmaceutical prescriptions identified by the Anatomical-Therapeutical-Chemical code), specialist appointments, diagnostic procedures, hospital admissions, growth parameters and outcome data of the children habitually seen by about 125 family pediatricians (FPs) distributed throughout Italy. The FPs participation in the database is voluntary and patients and their parents provided consent for use of their data for research purposes. In Italy each child is assigned to a FP, who is the referral for any health visit or any drug prescription, thus the database contains a very detailed personal medical history. The data, generated during routine patient care using common software (JuniorBit®), are anonymized and sent monthly to a centralized database in Padua for validation. For this study data relating to 120,338 children, including information related to 132,667 diagnoses and 1,595,842 drug prescriptions, from 12 Italian regions (Friuli-Venezia Giulia, Liguria, Lombardia, Piemonte, Veneto, Abruzzo, Lazio, Marche, Toscana, Campania, Sardegna, and Sicilia) were considered.

The study and the access to the database were approved by the Internal Scientific Committee.

### Study population and case identification

Information about patient characteristics, diagnosis, prescriptions, and test usage as well as rapid test results (positive, negative, and dubious) were obtained from the database for all identified cases of AOM or pharyngitis.

The study population included children aged from 0 months to 14 years with a primary ICD-9 code or descriptive diagnosis of acute otitis media (ICD-9-CM: 381.0, 381.00, 382, 382.0, 382.00 – “otite media acuta”), pharyngitis (034.0, 462- “faringotonsillite”), or tonsillitis (463- “tonsillite”). In order to avoid duplicates, medical records with the same diagnosis less than 30 days apart were considered as follow up of the initial case.

Pharyngitis diagnoses were divided in three groups based on FP diagnosis: GABHS pharyngitis, non-GABHS pharyngitis and non-defined pharyngitis. Strep-A rapid test was considered a suitable test to identify pharyngitis bacterial etiology. The test result was considered the gold standard for the diagnosis and all cases with a positive test result were classified as GABHS diagnosis, whereas negative results were classified as non-GABHS cases. Cases with dubious test results were classified according to the primary diagnosis.

Specific AOM exclusion criteria were: concomitant bacterial infections, ongoing antibiotic therapy, immunodeficiency or immunosuppressive therapy, tympanostomy tubes at the time of diagnosis, craniofacial abnormalities, chronic otitis media (381.1, 381.2, 381.3, 382.1, 382.2, 382.3), AOM complicated by mastoiditis (383), effusive otitis media, and chronic diseases (including cystic fibrosis and diabetes).

Specific pharyngitis exclusion criteria were: concomitant bacterial infections, ongoing antibiotic therapy, immunodeficiency or immunosuppressive therapy, previous tonsillectomy (28.2), chronic pharyngitis (472.1), chronic diseases (including cystic fibrosis and diabetes).

Ongoing antibiotic therapy was defined as antibiotic prescription in the 14 days before AOM or pharyngitis case.

For patients included in the study, only the first prescription per diagnosis was included.

## Statistical analysis

### AOM statistical analysis

Prescription distribution was described as the percentage of prescriptions per drug class (amoxicillin, amoxicillin and clavulanic acid (CV-Amoxicillin), II generation cephalosporins, III generation cephalosporins, macrolides, and other antibiotics).

We then calculated the frequency of prescriptions in each class according to age group (≤6 months, 6–24 months, and >  24 months, age bands used for treatment guidelines [23]) and trends in the prescription of antibiotics over time.

The “wait and see” approach was defined as all patients with AOM who did not receive antibiotic prescription within the first 48 h after diagnosis [33].

### Pharyngitis statistical analysis

Pharyngitis diagnoses were described according to (i) age (< 3 years, ≥3 years, age used for treatment guidelines [23]), (ii) test used to identify bacterial etiology, and (iii) results of the test. The frequency of prescriptions in each drug class was calculated by each of these strata as well.

## Results

We identified 120,338 children followed by 125 family pediatricians participating in Pedianet between 2010 and 2015. Among them 30,394 had at least one AOM diagnosis and 52,341 at least one pharyngitis diagnosis.

### AOM population

Among 30,394 children with AOM we recorded 54,943 distinct AOM diagnoses.

The mean age at diagnosis was 44 months, with 2% of children less than 6 months of age, 22% between 6 and 24 months and 76% older than 24 months of age.

In 45,320 (82.5%) AOM diagnoses the pediatrician prescribed an antibiotic within the 48 h from the diagnosis, while for 9623 cases (17.5%) the ‘wait and see’ approach was preferred. In 535 AOM initially treated with the ‘wait and see’ approach (535/9623, 5.6%), an antibiotic prescription was filled between 48 and 120 h after the diagnosis.

Amoxicillin and CV-Amoxicillin were the most frequently prescribed antibiotics overall (15,906/45320–35.1% vs 14,865/45320–32.8%), followed by III generation cephalosporins (9114/45320). Macrolides were less prescribed with a frequency of 3% (1347/45320) (Table 1).

**Table 1:** Distribution of first line antibiotic prescriptions for AOM with percentages referred to total prescription by age bands. Pedianet, Italy, 2010–2015

	Amoxicillin		CV-Amoxicillin		Cephalosporins - III gen.		Cephalosporins - I/II gen.		Macrolides/ Lincosamides		Other^a^		Total	
	(*N* = 15,906)		(*N* = 14,865)		(*N* = 9114)		(*N* = 4056)		(*N* = 1347)		(*N* = 32)		(*N* = 45,320)	
	N	(%)	N	(%)	N	(%)	N	(%)	N	(%)	N	(%)	N	(%)
≤6 months	486	(54.1)	259	(28.8)	78	(8.7)	63	(7.0)	13	(1.4)	0	(0)	899	(2.0)
6 mo. < age ≤ 24 mo.	4378	(44.6)	2630	(26.8)	1670	(17.0)	945	(9.6)	186	(1.9)	14	(0.14)	9823	(21.7)
> 24 months	11,042	(31.9)	11,976	(34.6)	7366	(21.3)	3048	(8.8)	1148	(3.3)	18	(0.05)	34,598	(76.3)

^a^ Clofoctol

**Fig. 1:**
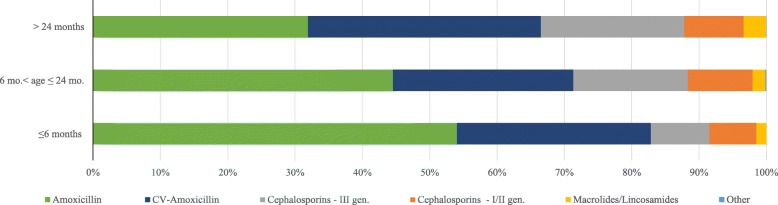
Distribution of first line antibiotic prescriptions for AOM differentiated by age bands. Pedianet, Italy, 2010–2015

The distribution of first line antibiotic therapy according to different age groups is described in Fig. 1 and Table 1. The use of amoxicillin seems to decrease with increasing age, while cephalosporins and CV-Amoxicillin prescriptions presented an opposite trend.

**Fig. 2:**
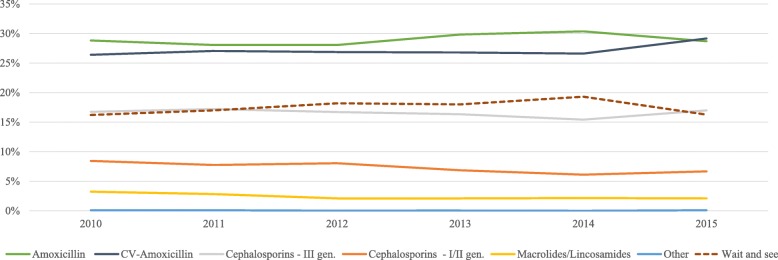
Distribution of first line treatment approach for AOM by years. Pedianet, Italy, 2010–2015

Indeed, the rate of CV-Amoxicillin prescriptions increased from 26.4% in 2010 to 29.2% in 2015, while the frequency of amoxicillin use and the ‘wait and see’ approach showed a slight increase until 2014 followed by a decrease in 2015. The frequency of other antibiotic use was constant over time (Fig. 2 with prescription rate provided in Table 2).

**Table 2:** Distribution of first line treatment approach for AOM with percentages referred to total treatments by years. Pedianet, Italy, 2010–2015

	Amoxicillin		CV-Amoxicillin		Cephalosporins - III gen.		Cephalosporins - I/II gen.		Macrolides/ Lincosamides		Other^a^		Wait and see	
	(*N* = 15,906)		(*N* = 14,865)		(*N* = 9114)		(*N* = 4056)		(*N* = 1347)		(*N* = 32)		(*N* = 9623)	
	N	(%)	N	(%)	N	(%)	N	(%)	N	(%)	N	(%)	N	(%)
2010	2868	(28.8)	2627	(26.4)	1667	(16.8)	840	(8.4)	323	(3.2)	9	(0.1)	1614	(16.2)
2011	2840	(28.1)	2738	(27.1)	1742	(17.2)	785	(7.8)	287	(2.8)	9	(0.1)	1720	(17.0)
2012	2748	(28.1)	2630	(26.9)	1636	(16.7)	787	(8.0)	205	(2.1)	2	(0)	1782	(18.2)
2013	2902	(29.8)	2606	(26.8)	1590	(16.3)	666	(6.8)	205	(2.1)	6	(0.1)	1752	(18.0)
2014	2549	(30.4)	2233	(26.6)	1295	(15.4)	513	(6.1)	181	(2.2)	0	(0)	1621	(19.3)
2015	1999	(28.7)	2031	(29.2)	1184	(17.0)	465	(6.7)	146	(2.1)	6	(0.1)	1134	(16.3)

^a^Clofoctol

### Pharyngitis population

Among 52,341 children, 126,098 distinct pharyngitis or tonsillitis diagnoses were recorded. 40.5% of them (51,144/126098) were recorded as non-defined pharyngitis, 30.1% (37,929/126098) as GABHS pharyngitis, and 29.4% (37,025/126098) as non- GABHS pharyngitis. The mean age was 5 years (IQ 3–8), with 76% (95,972/126098) of pharyngitis diagnosed after 3 years of age**.**

A rapid strep test was used in 34.8% of cases (43,927/126098), having a positive result in 56.8% of samples (10,299/18120 with 58.7% missing data regarding overall test results). Almost 86.2% (32,700/37929) of GABHS pharyngitis cases were diagnosed using the rapid strep test, whereas most of the non- GABHS pharyngitis were diagnosed without using the test (72.7% - 26,923/37025) (data not shown, see Additional file 1).

Overall, in 63.1% (79,620/126098) of pharyngitis an antibiotic was prescribed, 20.7% (16,466/79620) for non- GABHS pharyngitis, 43.5% (34,671/79620) for GABHS pharyngitis and 35.8% (28,483/79620) for non-defined pharyngitis (data not shown, see Additional file 2).

The 94.7% of pharyngitis with a positive test result (9749/10299) received a prescription, while for cases with a negative test result just 8.2% received an antibiotic prescription (605/7415).

As regarding diagnosis not confirmed by the test, an antibiotic was prescribed in 90.2% of GABHS pharyngitis (24,922/27630) and in 53.6% (15,861/29610) of non- GABHS pharyngitis.

Moreover, amoxicillin was prescribed in 55.8% (5438/9749) of pharyngitis with a positive test result versus CV-Amoxicillin and cephalosporins of III generation prescribed in 24% (2341/9749) and 11.6% (1127/9749), respectively. The probability of having prescribed amoxicillin or CV-Amoxicillin with a diagnosis of GABHS pharyngitis not confirmed by the test was 32.1% (8004/24922) and 16.1% (4022/24922), respectively (Table 3).

**Table 3:** Distribution of first line treatment antibiotic therapy for GABHS pharyngitis. Pedianet, Italy, 2010–2015

	GABHS pharyngitis					
	(*N* = 37,929)					
	No test- No result/Dubious result		Positive test		Total	
	N	(%)	N	(%)	N	(%)
Amoxicillin	10,602	(42.5)	5438	(55.8)	16,040	(46.3)
CV-Amoxicillin	8004	(32.1)	2341	[24]	10,345	(29.8)
Cephalosporins - III gen.	4022	(16.1)	1127	(11.6)	5149	(14.9)
Cephalosporins - II gen.	1349	(5.4)	389	[4]	1738	[5]
Macrolides/Lincosamides	925	(3.7)	449	(4.6)	1374	[4]
Other^a^	20	(0.1)	5	(0.1)	25	(0.1)
Total treated	24,922	(90.2)	9749	(94.7)	34,671	(91.4)
Total not treated	2708	(9.8)	550	(5.3)	3258	(8.6)

^a^Clofoctol

Patients diagnosed with non- GABHS pharyngitis, regardless of the strep test, received CV-Amoxicillin in 30.6% (4856/15861) of cases and cephalosporins of II and III/IV generation in about 11.6% (1835/15861) and 28.1% (4458/15861) of the diagnosis; the rate of amoxicillin prescription alone for non- GABHS diagnosis not confirmed by the test was 18.6% (2944/15861) (Table 4). Distribution of first line treatment antibiotic therapy for non-defined pharyngitis is reported in Additional file 3.

**Table 4:** Distribution of first line treatment antibiotic therapy for non-GABHS pharyngitis. Pedianet, Italy, 2010–2015

	non-GABHS pharyngitis					
	(*N* = 37,025)					
	No test- No result/Dubious result		Negative test		Total	
	N	(%)	N	(%)	N	(%)
Amoxicillin	2944	(18.6)	221	(36.5)	3165	(19.2)
CV-Amoxicillin	4856	(30.6)	149	(24.6)	5005	(30.4)
Cephalosporins - III gen.	4458	(28.1)	100	(16.5)	4558	(27.7)
Cephalosporins - II gen.	1835	(11.6)	28	(4.6)	1863	(11.3)
Macrolides/Lincosamides	1652	(10.4)	99	(16.4)	1751	(10.6)
Other^a^	116	(0.7)	8	(1.3)	124	(0.8)
Total treated	15,861	(53.6)	605	(8.2)	16,466	(44.5)
Total not treated	13,749	(46.4)	6810	(91.8)	20,559	(55.5)

^a^Clofoctol

## Discussion

This study provides interesting insight into paediatric antibiotic prescriptions at the primary care level in Italy. Only a few reports on antibiotic prescriptions for pediatric populations have been published and even fewer linking antibiotic prescription to the diagnosis in primary care settings [34, 35].

In particular, the possibility to link antibiotic prescriptions with the related diagnosis in a longitudinal dataset is helpful for tracking prescribers’ attitudes and understanding prescription trends over time.

In keeping with the literature, the pediatric population with AOM included in our study had a mean age of 3–4 years (44 months), with 22% children between 6 and 24 months and only 2% younger than 6 months of age.

Despite the mean age of the population analyzed the “wait and see” was adopted only in 18.5% of the cases as previously noted in other studies conducted in the hospital setting [36, 37].

However, we were unable to identify the use of delayed prescription approach where the physician provides an antimicrobial prescription at the time of diagnosis but asks parents to wait 48 to 72 h and administer the antibiotic only if there is no improvement in symptoms [29, 38].

An increase in broad spectrum prescriptions has been associated with the increase of children’s age; more than half of the prescriptions for children older than 24 months in our study were for broad spectrum antibiotics. In contrast, more than 60% of children younger than 6 months received narrow spectrum antibiotics. This attitude may derive from the fear of beta lactamase producing bacterial infections for children over 24 months of age, possibly acquired in crowded places such nursery schools, or to the uncertainty of possible cumulative resistance in this age group after previous treatment with narrow-spectrum antibiotics. This prescribing attitude has also been reported in hospital settings and reflects prescription data from other pharmacoepidemiological studies [15].

The general trend towards broad spectrum antibiotic prescription as first line treatment was also reflected in the annual analysis where it was clear that at the end of the period analyzed (2015), after a previous slight increment in amoxicillin prescriptions, the guideline recommended first line approaches rate (together with ‘wait and see’ approach) decreased.

As regarding pharyngitis, our analysis found that the test was used mostly in GABHS diagnosis compared to non- GABHS ones, maybe due to the fact that pediatricians used this tool to confirm the empirical bacterial etiology instead of excluding it. This demonstrates apparent trust in clinical diagnosis by treating physician.

However, the fact that more antibiotics, mostly broad-spectrum (CV-Amoxicillin and III gen. cephalosporin), were prescribed for a clinical diagnosis of GABHS pharyngitis indicates a more cautious interpretation of the clinical symptom evaluation.

Moreover, children with rapid test confirmed GABHS pharyngitis received mostly narrow-spectrum antibiotics (amoxicillin) while those without test results were equally likely to receive narrow or broad-spectrum treatment.

Almost 5% of the children with rapid test positive GABHS pharyngitis were not treated with antibiotics. These results are comparable with the European population of pediatric GABHS carriers found in the literature [32].

These results seemed to confirm once again that diagnostic uncertainty is one of the determinants for overall antibiotic over prescription [39], together with perceived parental expectations of an antibiotic prescription and fear of under treatment as previously found in other studies [40].

Focusing more deeply on the antibiotics prescribed it is worrisome realizing that III generation cephalosporins are so highly prescribed for non- GABHS pharyngitis whether or not confirmed by the test (22% vs 28% of total prescriptions for not confirmed and confirmed, respectively). These outcomes seemed to reflect a trend already known in the hospital setting [14] both in pediatric and adult populations [41], confirming an abuse of these antibiotics. However, it should be considered that, despite the initial pharyngitis diagnosis, the pediatrician could have prescribed an antibiotic suspecting another upper or lower respiratory tract infection.

Another analysis conducted by de Bie et al. [42], confirmed the overuse of antibiotics in the Italian pediatric population, compared especially with north European countries; even though amoxicillin remains the most prescribed antibiotic, the prevalence of broad spectrum prescriptions in Italy was almost double compared to the United Kingdom and the Netherlands where amoxicillin prescriptions accounted for 50% of antibiotic prescriptions. These results are in line with our findings especially for AOM and non-GABHS pharyngitis. Furthermore, an 8-year survey conducted in all hospitals in the Emilia-Romagna Region showed a steadily increasing consumption of broad-spectrum antibiotics as noted in our study for AOM prescriptions and a considerable decrease of narrow-spectrum ones [43].

As for the outpatient population, according to drug prescriptions dispensed during 2006 by some retail pharmacies in Italy, 52% of the paediatric population received at least one antibiotic therapy, a little bit more for males and less for females [4]. It has been estimated that almost half of antibiotics prescribed by a primary care physician are unnecessary [44] since the majority of them are prescribed for common pediatric infections such as pharyngitis (considered also in our analysis) that mainly have a viral cause.

For the aforementioned reasons it is imperative to find a way to reduce antibiotics use, particularly in pediatric populations.

As regarding AOM and pharyngitis few regions implemented guidelines for the diagnosis and the treatment of these illnesses (e.g. Veneto region implemented the “Cure Primarie” project in 2006) and some consensus and guidelines written both by pediatric ENT specialist and pediatric infectious diseases specialist have been published in the last decades [22, 23, 30].

As our results point out, guidelines do not seem to be the most suitable tool in primary care settings where the need of rapid decision making limit the consultation of lengthy guidelines.

Interestingly, Gerber et al. [45] proved that clinician education coupled with audit and feedback of prescribing significantly improved antibiotic use for outpatient children; the overall proportion of broad-spectrum antibiotic prescriptions decreased from 26.8 to 14.3% after the intervention. Although very effective, these two core antibiotic stewardship (AS) interventions are expensive (median cost $187.400) [46] and this could limit their implementation in the Italian healthcare setting.

On the other hand, other AS interventions such as clinical pathways (CP) could represent useful and practical evidence-based tools to guide antibiotic prescribing where both personnel and economic resources are restricted. In Italy clinical pathways for pharyngitis, AOM and community acquired pneumonia were successfully implemented in a tertiary care hospital with a significant reduction of broad spectrum antibiotic prescriptions and of total antibiotic cost [17, 47].

The Pedianet database or other similar data sources could be valuable tools to measure the effectiveness and costs of AS interventions as well as other healthcare policy actions. The possibility of accessing data related to the daily activities of FP is a unique resource, both for studying individual diseases, as well for pharmacoepidemiological and pharmacoeconomical analysis. Pedianet is an example of an efficient pediatric outpatient network collecting specific data from computerized clinical files. With more than 300 Italian pediatricians enrolled throughout the country, this network has been proven to be able to carry out epidemiological studies as well as pharmacovigilance ones [48–50].

Our study had several limitations including the retrospective nature of the analysis and the lack of manual evaluation and validation of the diagnosis potentially including false positive cases in the analysis.

Secondly, as mentioned earlier the fact that we cannot identify the delayed prescription for AOM is one limitation.

As regarding rapid strep usage not all clinicians have the possibility to perform the test in their practice, thus this could represent a bias.

Finally, with our data it was not possible to separate pediatricians who might have received an educational training on treatment of the diagnosis considered that may have changed their practice through the years. On the other hand, the high number of FP enrolled and the variety of settings of their practices (urban, suburban, rural) is a positive element in supporting the generalizability of results.

## Conclusion

The analysis conducted using a large Italian family pediatricians’ database confirmed the increasing prescriptions rate of broad-spectrum antibiotics (especially amoxicillin/clavulanate and III generation cephalosporins) for AOM and pharyngitis already observed in the literature. With the increasing concern about antimicrobial resistance, the identification of potential barriers for successful intervention remains crucial, especially where resources are limited. Future studies should examine the best approach to guide pediatric antibiotic prescribing in Italian outpatient settings where challenges are represented mainly by high rates of patient turnover and rapid decision-making, usually without microbiology support.

## Additional files

Additional file 1: Distribution of test results according to pharyngitis diagnosis. Pedianet, Italy, 2010–2015. (XLSX 9 kb)

Additional file 2: Distribution of first line approach according to pharyngitis diagnosis. Pedianet, Italy, 2010–2015. (XLSX 9 kb)

Additional file 3: Distribution of first line treatment antibiotic therapy for non-defined pharyngitis. Pedianet, Italy, 2010–2015. (XLSX 9 kb)

## Data Availability

The datasets generated during and/or analyzed during the current study are available from the corresponding author on reasonable request.

## Abbreviation

AOM: Acute Otitis Media
AS: Antibiotic Stewardship
CP: Clinical Pathways
CV-Amoxicillin: Amoxicillin and Clavulanic Acid
ENT: Ear Nose and Throat
FIMP: Federazione Italiana Medici Pediatri
FP: Family Pediatrician
GABHS: *Group A β-hemolytic streptococcus*
ICD-9: 9th International Statistical Classification of Diseases and Related Health Problems system
IQ: Inter-Quartile
SIP: Società Italiana di Pediatria
